# Ultralow Overpotential in Rechargeable Li–CO_2_ Batteries Enabled by Caesium Phosphomolybdate as an Effective Redox Catalyst

**DOI:** 10.1002/advs.202502553

**Published:** 2025-04-30

**Authors:** Mahsa Masoudi, Neubi F. Xavier Jr, James Wright, Thomas M Roseveare, Steven Hinder, Vlad Stolojan, Qiong Cai, Robert C. T. Slade, Daniel Commandeur, Siddharth Gadkari

**Affiliations:** ^1^ School of Chemistry and Chemical Engineering Faculty of Engineering and Physical Sciences University of Surrey Guildford GU2 7XH UK; ^2^ Department of Chemistry University of Sheffield Brook Hill Sheffield S3 7HF UK; ^3^ School of Mechanical Engineering Sciences Faculty of Engineering and Physical Sciences University of Surrey Guildford GU2 7XH UK; ^4^ Advanced Technology Institute School of Computer Science and Electronic Engineering Faculty of Engineering and Physical Sciences University of Surrey Guildford GU2 7XH UK

**Keywords:** caesium phosphomolybdate, electrocatalysts, lithium‒CO_2_ batteries, polyoxometalates, stability, ultralow overpotential

## Abstract

Rechargeable lithium‐CO_2_ batteries are emerging as attractive energy storage devices due to their potential for high capacity and efficient CO_2_ reduction, making them promising candidates for post‐lithium‐ion batteries with high energy densities. However, their practical applications have been restricted by low reversibility, poor cycle life, and sluggish redox kinetics induced by the high potential required for decomposing the discharge product Li_2_CO_3_. Despite the various cathode catalysts explored, their application is often limited by availability, high cost, and complexity of synthesis. Herein, caesium phosphomolybdate (CPM) is synthesized through a facile and low‐cost method. The Li‒CO_2_ battery based on the CPM cathode demonstrates a high discharge capacity of 15 440 mAh g^−1^ at 50 mA g^−1^ with 97.3% coulombic efficiency. It further exhibits robust stability, operating effectively over 100 cycles at 50 mA g^−1^ with a capacity limitation of 500 mAh g^−1^. Remarkably, the CPM catalyst yields a low overpotential of 0.67 V, surpassing most catalysts reported in prior research. This study reports, for the first time, the application of a Keggin‐type polyoxometalate as a bifunctional redox catalyst, significantly improving the reversible cycling of rechargeable Li–CO_2_ batteries.

## Graphical abstract

This study represents the first application of a Keggin‐type polyoxometalate, caesium phosphomolybdate (CPM), as a bifunctional redox catalyst for rechargeable Li–CO_2_ batteries. CPM significantly reduced the potential gap between discharge and charge reactions to 0.67 V, outperforming most reported catalysts. It also exhibited a high discharge capacity with robust stability over 100 cycles.

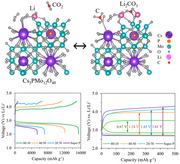

## 1 Introduction

The ongoing emissions of CO_2_, primarily driven by the excessive consumption of fossil fuels, have led to significant challenges for global climate stability and ecological preservation.^[^ ^1^, ^2^ ^]^ Numerous efforts have been dedicated to developing sustainable technologies that effectively reduce CO_2_ concentration by capturing and converting it into valuable fuels and chemicals.^[^ ^3^, ^4^, ^5^ ^]^ To this end, rechargeable lithium‐CO_2_ (Li–CO_2_) batteries utilizing CO_2_ gas as an energy carrier, present an attractive approach for not only CO_2_ reduction but also energy conversion and storage to obtain a net‐zero economy and global sustainability.^[^ ^6^, ^7^ ^]^ Li–CO_2_ batteries have recently emerged as potential alternatives to conventional lithium‐ion batteries (LIBs)^[^ ^8^ ^]^ owing to their superior theoretical energy density (1876 Wh kg^−1^) and high discharge voltage (≈2.8 V) based on the electrochemical reaction of 3CO_2_ + 4Li^+^ + 4e^−^ ↔ 2Li_2_CO_3_ + C.^[^ ^9^ ^]^ Moreover, the capability of Li–CO_2_ batteries to operate in a CO_2_‐rich environment makes them promising candidates for supplying sustainable energy for extended interplanetary Mars exploration (where 96% of the atmosphere is CO_2_) or underwater missions.^[^ ^10^, ^11^ ^]^

Despite the promise of Li–CO_2_ batteries for CO_2_ conversion and energy storage, several technical challenges have impeded their commercialization and practical application.^[^ ^12^ ^]^ Since a high voltage (> 4 V) is required to drive the reversible charge reaction for the complete decomposition of the wide‐bandgap lithium carbonate (Li_2_CO_3_),^[^ ^13^ ^]^ the discharge products (Li_2_CO_3_ and carbon) are prone to aggregate on the electrode‐electrolyte interface. This can result in large electrode impedance, high charging voltage, lithium dendrite formation, and sluggish redox kinetics.^[^ ^14^ ^]^ This leads to serious challenges such as poor reversibility, low capacity, short cycle life, and safety concerns.^[^ ^15^ ^]^ Therefore, the design and development of highly efficient heterogeneous electrocatalysts are needed to enhance the kinetics of the CO_2_ reduction/evolution reactions (CRR/CER) and mitigate the high overpotential between CRR and CER during cycling.^[^ ^16^ ^]^

Although many types of cathode catalysts have been utilized in Li–CO_2_ batteries so far,^[^ ^17^ ^]^ their real‐world application is mostly hampered by limited availability, high cost, and complicated synthesis. For example, noble metals (NMs) such as ruthenium^[^ ^18^, ^19^, ^20^ ^]^ and iridium^[^ ^21^, ^22^, ^23^ ^]^ have exhibited remarkable catalytic activity in improving round‐trip efficiency and lowering the overpotential of Li–CO_2_ batteries. However, substantial costs, complex synthesis, and easy agglomeration of monometallic nanoparticles limit their practicability.^[^ ^24^ ^]^ Meanwhile, transition metals (TMs) and their compounds are regarded as promising alternatives due to their relative availability, low cost, polyvalent characteristics, tunable d‐band centers, and rich electron d orbitals.^[^ ^25^, ^26^, ^27^ ^]^

Phosphomolybdic acid (PMA) is a well‐known Keggin‐type heteropolyacid from the polyoxometalates (POMs) family.^[^ ^28^ ^]^ POMs are polyatomic anions consisting of transition metal (groups 5 or 6) oxyanions interconnected via shared oxygen atoms.^[^ ^29^ ^]^ PMA is defined as a metal‐oxo cluster, which consists of 12 octahedral oxo‐bridged (oxo‐ligated) molybdenum atoms in their highest oxidation state (VI) surrounding a central phosphate group, building a 3D framework.^[^ ^30^ ^]^ This 3D unique electronic structure with an oxygen‐rich surface endows PMA with a significant redox capability, which can drive multi‐electron redox reactions without altering the PMA structure.^[^ ^31^ ^]^ This highly negatively charged framework makes PMA a superb electron acceptor and promising catalyst candidate, which can effectively facilitate redox kinetics.^[^ ^32^ ^]^ However, a major drawback of PMA is its low surface area, which restricts its electrocatalytic activity.^[^ ^33^ ^]^ Integrating alkali metals can greatly improve the structural and physicochemical properties of PMA.^[^ ^34^ ^]^ PMA intercalated with monovalent large‐size cations such as Cs^+^, K^+^, and Rb^+^ displays high surface area and notable changes in pore size.^[^ ^35^ ^]^ Another disadvantage of the parent PMA is its high solubility in water. To solve this problem, acidic clusters are stabilized and made insoluble by interactions with alkali metal ions such as Cs^+^, synthesizing partially saturated solid salts.^[^ ^36^ ^]^ To the best of our knowledge, the application of POM‐based materials as potential catalysts in the research field of rechargeable metal‐CO_2_ batteries has not been reported thus far.

Herein, caesium phosphomolybdate (Cs_3_PMo_12_O_40_, CPM) was applied as a catalyst in Li‒CO_2_ batteries for the first time. The Keggin POM nanocomposite was prepared via a simple and low‐cost solid‐state method conducted at room temperature. The prepared CPM cathode catalyst in a Li–CO_2_ battery delivered a high discharge capacity of 15440 mAh g^−1^ at a current density of 50 mA g^−1^ and a long stability of 107 cycles at 50 mA g^−1^ with a limited capacity of 500 mAh g^−1^. Interestingly, the battery displayed a low overpotential of 0.67 V, surpassing the performance of many known cathode catalysts for Li‒CO_2_ batteries, particularly those composed of noble metals. The outstanding characteristics of CPM including abundant electroactive sites, oxygen‐enriched surface, and mesoporous morphology make it a stable and efficient catalyst, that enhances the CRR and CER kinetics during discharge‐charge cycling of Li–CO_2_ batteries. The CPM pore structure formed by small particle size (≈140 nm) not only allows fast diffusion of CO_2_ molecules and Li^+^ ions to the active sites but also provides adequate pores volume for the accommodation of discharge products. In summary, the synthesized CPM presented an excellent bi‐functional catalyst, offering a higher discharge‐charge capacity and lower overpotential batteries than comparable materials in previous literature.

## 2 Results and Discussion

### 2.1 Structural and Morphological Characterizations of the CPM Catalyst

**Figure 1:**
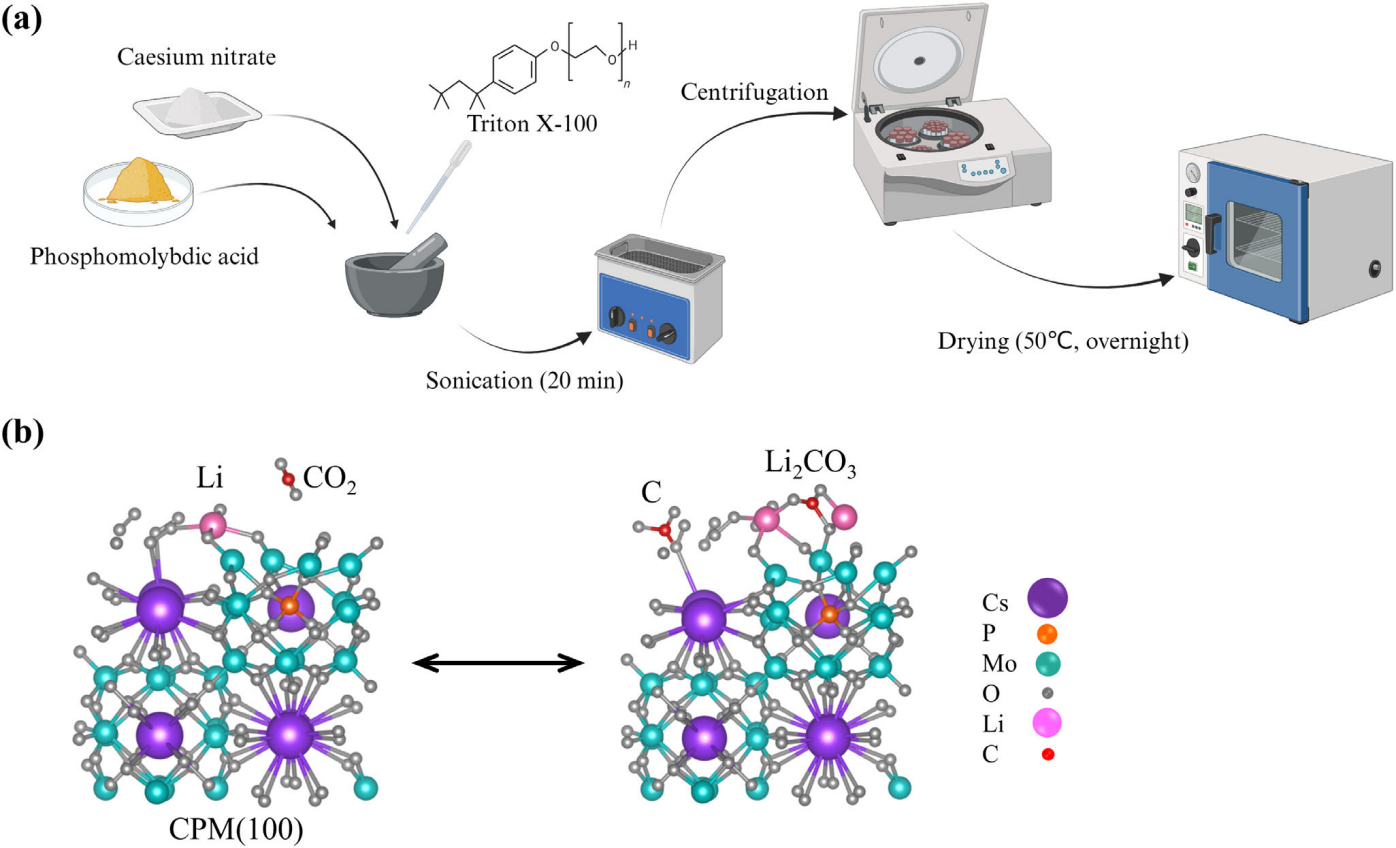
Schematic representation of a) the CPM synthesis process and b) its catalytic reaction pathway in a Li–CO_2_ battery.

**Figure 2:**
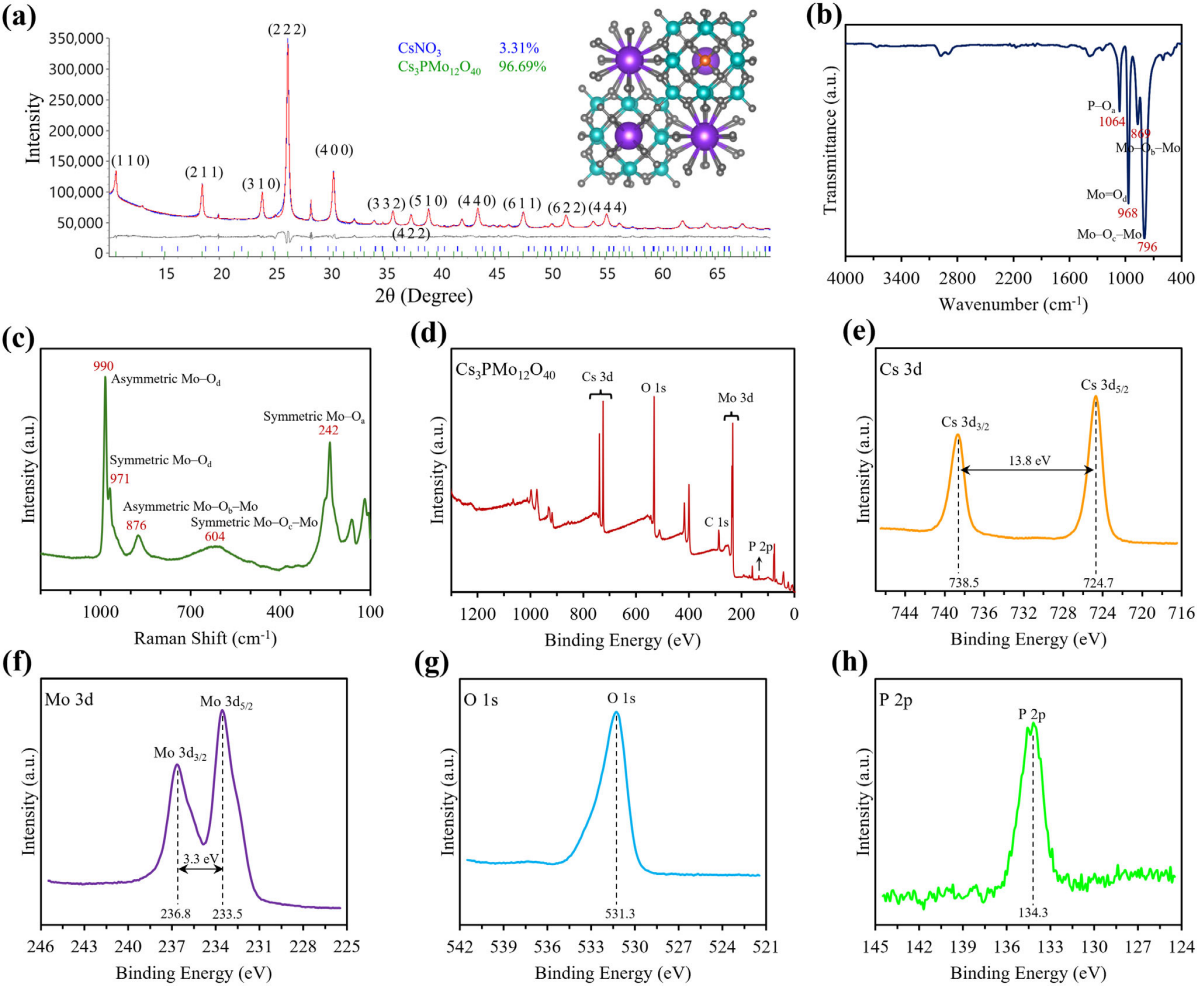
Material characterization of synthesized CPM measured by a) PXRD data: observed (blue) and calculated (red) profiles and difference plot (I_obs_ – I_calc_) (grey) of the mixed‐phase Rietveld refinement (2θ = 10–70°, d_min_ = 1.34 Å); inset displays the side view of CPM structure: purple, Cs; blue, Mo; orange, P; grey, O., b) FTIR spectroscopy, and c) Raman spectroscopy. XPS analysis of prepared CPM catalyst: d) survey spectrum, and high‐resolution XPS spectra of e) Cs 3d, f) Mo 3d, g) O 1s, and h) P 2p.

CPM catalyst was synthesized through a facile solid‐state method at room temperature (**Figure** **1**a)^[^ ^37^ ^]^ and employed in a Li‒CO_2_ battery (Figure 1b). The crystal lattice structure and composition of the as‐synthesized CPM catalyst were confirmed by powder X‐ray diffraction (PXRD) (**Figure** **2**a). The PXRD profile of CPM arises from a body‐centered cubic (BCC) lattice composed of Keggin units, with prominent diffraction peaks at 10.59°, 18.40°, 23.83°, 26.14°, and 30.27° corresponding to (1 1 0), (2 1 1), (3 1 0), (2 2 2), and (4 0 0) crystal planes, respectively (JCPDS 046–0481). The PXRD pattern of peaks closely matches that reported by Bykhovskii et al.,^[^ ^38^ ^]^ confirming the successful synthesis of the CPM catalyst using a cost‐effective, one‐pot solid‐state technique. The unit cell of the CPM catalyst material was determined using TOPAS^[^ ^39^, ^40^ ^]^ (Figure S1, Supporting Information) and compared with published polyoxometalate crystal structures. As no crystal structure of the Cs_3_PMo_12_O_40_ material was available, a structural model of the [PMo_12_O_40_]^3−^ cluster was adapted from the published crystal structure of H_3_PW_12_O_40_,^[^ ^41^ ^]^ which crystallized in the same space group and similar cubic unit cell. Waters of crystallization/hydroxonium ions from the published H_3_PW_12_O_40_ structure were replaced at fractional coordinates [0.75, 0.25, 0.25] with Cs atoms. A mixed‐phase Rietveld analysis^[^ ^42^ ^]^ indicated a crystalline material composition of 96.69% Cs_3_PMo_12_O_40_, 3.31% CsNO_3_ [Cs_3_PMo_12_O_40_ (Cubic, *Pn‐3* *m*): a = 11.7770 (5) Å; CsNO_3_ (Hexagonal, *P3_1_*) a = 10.92 (3) Å, c = 7.72 (4) Å].

To further verify the presence of the Keggin ion in the CPM particles, Fourier transform infrared (FTIR) and Raman spectroscopies were employed. In the Keggin anion structure of the CPM (PMo_12_O_40_^3−^), a central PO_4_ tetrahedron is enveloped by four Mo_3_O_13_ clusters. Each Mo_3_O_13_ unit is composed of three Mo atoms coordinated with oxygen atoms, forming edge‐sharing octahedra. These Mo_3_O_13_ units are connected to each other and to the PO_4_ tetrahedron by corner‐sharing oxygen atoms, producing four distinct types of oxygen that have specific vibrational modes in the fingerprint region from 700 to 1200 cm^−1^.^[^ ^43^ ^]^ This structure is evidenced in Figure 2b, the recorded IR spectrum of the CPM displays asymmetric stretching vibrations of P–O_a_, Mo = O_d_, Mo–O_b_–Mo, and Mo–O_c_–Mo bonds within the Keggin structure at 1064, 968, 869, and 796 cm^−1^, respectively.^[^ ^44^ ^]^ The subscripts assigned to oxygen atoms indicate their specific locations within the CPM cluster. Moreover, the Raman spectrum presents the main characteristic bands of the Keggin structure at 990, 971, 876, 604, and 242 cm^−1^, which are assigned to asymmetric Mo–O_d_, symmetric Mo–O_d_, asymmetric Mo–O_b_–Mo, symmetric Mo–O_c_–Mo, and symmetric Mo–O_a_ (with bridge stretching character), respectively (Figure 2c). The Raman observation aligned with prior published results,^[^ ^45^, ^46^ ^]^ validating FTIR investigation and the presence of the Keggin unit in the synthesized catalyst. The structural analysis confirms that the CPM catalyst consists of a well‐defined BCC lattice composed of Keggin units. This crystalline framework provides high structural stability and maintains the catalyst's integrity over long‐term cycling.

X‐ray photoelectron spectroscopy (XPS) was used to identify the surface elemental composition and chemical states of CPM electrocatalyst. Figure 2d shows the XPS survey spectrum of CPM, which confirms the presence of P, Mo, O, and Cs atoms at their binding energy positions. In addition, Figure S2a–c (Supporting Information) displays that the Cs/Mo ratio remains consistent across three analyzed regions, highlighting the uniformity of the surface composition. In the core‐level spectrum of Cs 3d, two peaks were observed at 724.7 and 738.5 eV corresponding to Cs 3d_5/2_ and Cs 3d_3/2_, respectively (Figure 2e), validating the presence of Cs atom in Cs (+1) oxidation state. As shown in Figure 2f, the high‐resolution XPS spectrum of Mo 3d illustrates a doublet corresponding to Mo 3d_5/2_ and Mo 3d_3/2_ peaks located at 233.5 and 236.8 eV, respectively, revealing that Mo is primarily in the +6 oxidation state. Additionally, peak fitting of the Mo 3d peaks in Figure S2d‒f (Supporting Information) verifies that Mo^6+^ remains the dominant oxidation state, with a small amount of Mo^5+^ present and no significant variations in the oxidation state of Mo across different regions of the catalyst surface. The abundance of Mo^6+^ species in the CPM structure plays a crucial role in promoting CRR by acting as electron acceptors that adsorb and activate CO_2_. They weaken C = O bonds, stabilize intermediates, and lower activation energy, thereby facilitating electron transfer and improving CRR efficiency. In high‐resolution XPS spectra of oxygen (Figure 2g) and phosphorus (Figure 2h), intense peaks corresponding to O 1s at 531.3 eV and P 2p at 134.3 eV are evident, indicating valence states of O (‐2) and P (+5), respectively.^[^ ^47^ ^]^

In addition, the water content of the CPM composite was determined by thermogravimetry (TG) (Figure S3, Supporting Information). The thermogravimetric profile of mass loss of the sample over the temperature range of 30‒400 °C displays that the dehydration process started below 100 °C and continued until ≈400 °C. The total mass loss was 10.5%, which equates to 13 moles of water per mole of CPM, consistent with the reported values in the literature (9‒14 water moles) depending on the chosen method and total drying time.^[^ ^48^ ^]^ Figure S4a (Supporting Information) presents the N_2_ adsorption‐desorption isotherm of the CPM catalyst with the corresponding pore size distribution (Figure S4b, Supporting Information) to study its surface area and porosity. According to the IUPAC classification, the N_2_ adsorption‐desorption curve reveals a typical IV isotherm with an obvious H3‐type hysteresis loop.^[^ ^30^, ^49^ ^]^ The presence of a hysteresis loop confirms the mesoporous structure of the CPM catalyst,^[^ ^50^ ^]^ which can significantly facilitate the diffusion of CO_2_/Li^+^ ions and improve the catalytic CRR/CER performance. The mesoporous CPM displayed a specific surface area of 10.3 m^2^ g^−1^ with a pore volume below 0.04 cm^3^ g^−1^. The pore size distribution was measured by Barrett‐Joyner‐Halenda (BJH) method using the desorption branch,^[^ ^51^ ^]^ yielding an average pore diameter of 17.7 nm. The porous morphology of the CPM ensures sufficient pore volume to accommodate the discharge products, which consequently enables higher capacities.

**Figure 3:**
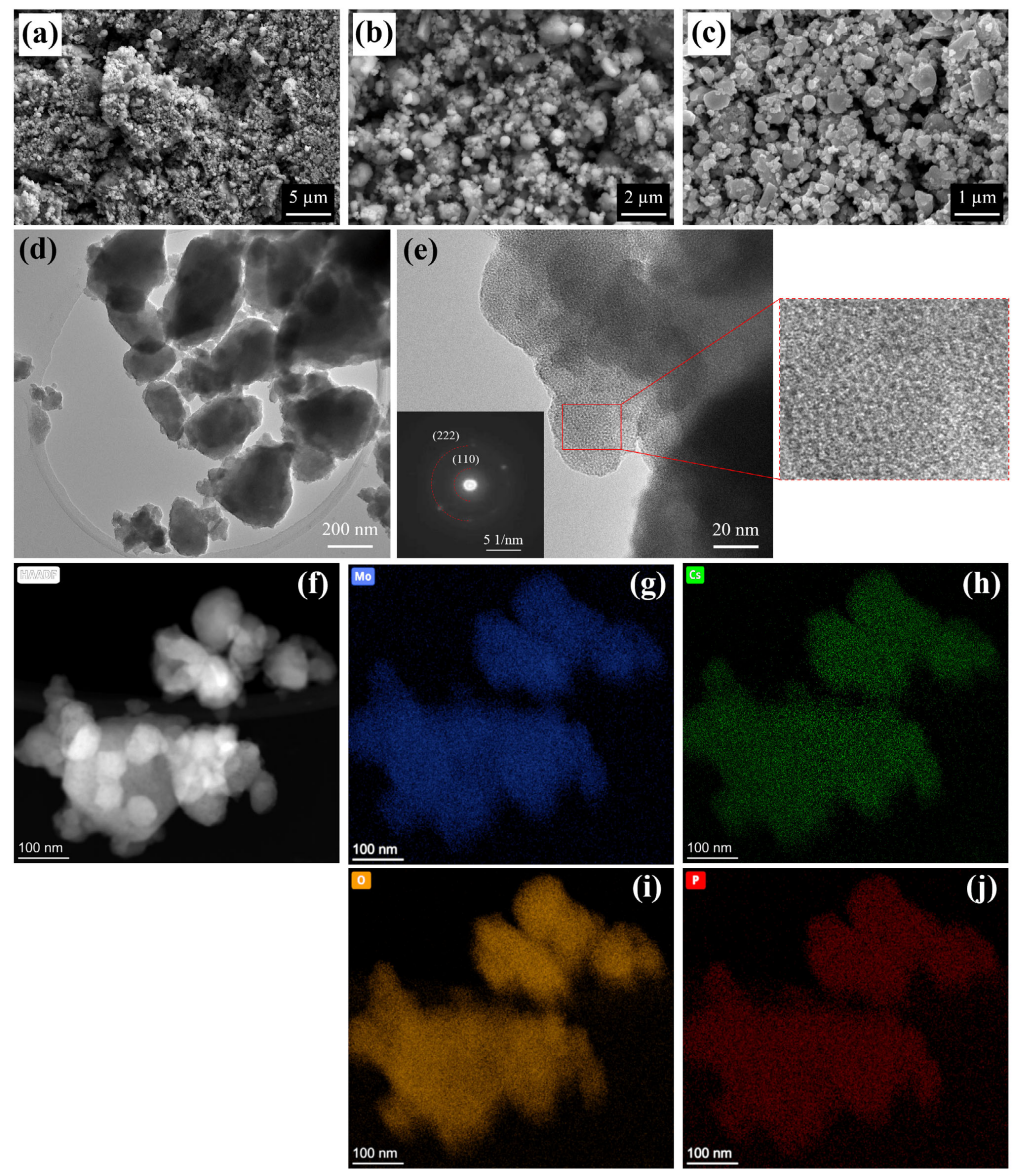
a‒c) SEM images of the as‐prepared CPM catalyst at different magnifications and an acceleration voltage of 10 kV, d,e) HRTEM images of CPM with the corresponding SAED pattern (inset), and f) HAADF‐STEM image of the CPM with the corresponding elemental mapping of Mo, Cs, O, and P g–j).

The surface morphology of the catalyst was characterized by scanning electron microscopy (SEM). SEM images of the CPM particles at different magnifications are presented in **Figure** **3**a‒c, indicating a porous interconnected network of aggregated spherical particles with a mean particle size of 140±62 nm. Validating the SEM observations, transmission electron microscopy (TEM) demonstrates a sphere‐like morphology for the CPM (Figure 3d) with periodic lattice fringes observed by high‐resolution TEM (HRTEM) in Figure 3e, suggesting the ordered, crystalline nature of CPM nanoparticles. The inset in Figure 3e exhibits distinct diffraction rings in the selected area electron diffraction (SAED) pattern of CPM. The two visible diffraction rings can correspond to (1 1 0) and (2 2 2) planes of CPM with spacings x and y, further revealing the polycrystalline characteristic of the CPM catalyst, which aligns with the structure obtained from PXRD results. Furthermore, active phase distribution over the CPM surface was analyzed by energy dispersive spectroscopy (EDS) and elemental mapping. As shown in Figure S5 (Supporting Information), the EDS spectrum of the as‐synthesized CPM verified the presence of Cs, Mo, O, and P elements. Additionally, the high‐angle annular dark field scanning transmission electron microscopy (HAADF‐STEM) image with EDS elemental mapping displayed that the four main elements are uniformly distributed across the CPM surface (Figure 3f‒j), underscoring the successful synthesis and homogeneity of the sample.

The morphological characterization highlights a mesoporous structure, which facilitates the diffusion of CO_2_ and Li^+^ ions, reduces mass transport limitations, and enhances catalytic activity. Brunauer‐Emmett‐Teller (BET) analysis further supports this by showing a hierarchical pore system that accommodates discharge products, leading to higher discharge capacities.

### 2.2 Electrochemical Performance of CPM‐based Cathodes in Li‒CO_2_ Batteries

In order to evaluate the electrocatalytic capability of the CPM catalyst in enhancing CRR/CER kinetics, Li‒CO_2_ batteries were assembled with CPM‐based cathodes. First, cyclic voltammetry (CV) of the cathodes assembled in the batteries was performed between 2.0 and 4.4 V at a sweep rate of 0.5 mV s^−1^ under CO_2_.

**Figure 4:**
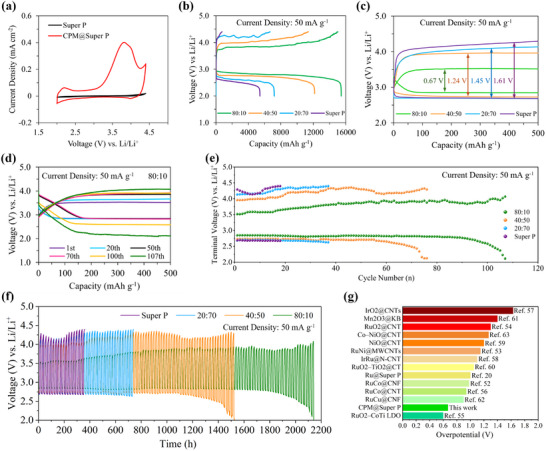
a) CV curves of Super P and 80:10 CPM cathodes, b) full GCD profiles and c) overpotential plots of Li‒CO_2_ batteries with 80:10, 40:50, 20:70 and Super P cathodes at the current density of 50 mA g^−1^. d) Long‐term cycling of the Li‒CO_2_ battery using the 80:10 CPM cathode at the current density of 50 mA g^−1^ and limited capacity of 500 mAh g^−1^. e) End discharge/charge potential over cycling and f) overall cycling duration of 80:10, 40:50, 20:70 and Super P cathodes in Li‒CO_2_ batteries at 50 mA g^−1^. g) Overpotential comparison of the optimum composition (80:10 CPM cathode) used in the current study with reported catalysts.

As revealed in **Figure** **4**a, the CV profile of the CPM cathode (80:10) showed a cathodic peak starting at ≈2.7 V corresponding to CO_2_ reduction. Over the anodic scan, a strong anodic peak appeared at ≈3.8 V due to the electrochemical oxidation of the discharge products. Compared to the Super P cathode, the CPM cathode displayed a higher onset voltage (≈2.7 V) toward the CRR and a lower onset voltage (≈3.2 V) for the CER, demonstrating the excellent ability of the CPM electrocatalyst to kinetically activate both reactions during battery cycling. Moreover, the CPM cathode suggested higher peak currents than the Super P cathode, which confirms the notable capability of the bifunctional CPM catalyst to accelerate the CRR/CER kinetics and lower the discharge‐charge polarization.

Figure 4b presents the full galvanostatic charge‐discharge (GCD) measurements of the CPM‐based batteries compared to the Super P cathode at a current density of 50 mA g^−1^ within a limited potential range of 2.0–4.4 V. CPM cathodes with varying catalyst‐to‐Super P ratios (90:0, 80:10, 40:50, and 20:70) were also compared to investigate the impact of the CPM catalyst amount on battery performance. Among them, the Li‒CO_2_ battery with the 80:10 CPM cathode delivered the highest specific discharge capacity of 15 440 mAh g^−1^, which is 2.8 times larger than that of the Super P cathode (5420 mAh g^−1^). The 80:10 cathode could effectively drive the reversible charge reaction, yielding an excellent coulombic efficiency (CE) of 97.3%. However, the Super P cathode demonstrated poor reversibility with a very low CE of 13.8%. Additionally, 40:50 and 20:70 cathodes produced discharge capacities of 12164 and 8394 mAh g^−1^, respectively, which are 2.2 and 1.5 times higher than that of the Super P cathode. The 90:0 CPM‐based cathode (without Super P) exhibited a discharge capacity of 9918 mAh g^−1^ with a CE of 84%, further demonstrating the catalytic contribution of CPM alone (Figure S6a, Supporting Information). These results further confirm the satisfactory catalytic activity of the proposed catalyst, as even a 40% incorporation can double the discharge capacity of the Li‒CO_2_ battery. The significant improvement in the discharge capacity and CE highlights the advantages of the CPM as a promising bifunctional cathode electrocatalyst for Li‒CO_2_ batteries. The mesoporous interconnected structure of the catalyst provides ample pore volume for Li_2_CO_3_ deposition, thereby leading to high discharge capacities. Furthermore, the high CE values produced by CPM‐assembled batteries suggested that CPM could effectively facilitate the reversible reaction between discharge products thanks to its oxygen‐rich surface and abundant active sites.

CPM also showed excellent electrocatalytic activity, improving the CRR/CER kinetics and mitigating the overpotential as presented in Figure 4c. In the full GCD test, the battery with the 80:10 cathode had a very low charge plateau of 3.5 V with a stable discharge plateau of ≈2.8 V (close to the theoretical discharge potential of Li‒CO_2_ batteries). Interestingly, the 80:10 cathode exhibited an ultra‐low overpotential of 0.67 V, which outperforms very nearly all of the known cathode electrocatalysts for Li‒CO_2_ batteries, especially those based on noble metals such as Ru and Ir^[^ ^20^, ^52^, ^53^, ^54^, ^55^, ^56^, ^57^, ^58^, ^59^, ^60^, ^61^, ^62^, ^63^ ^]^ (Figure 4g and Table S1, Supporting Information). Although the RuO_2_‒CoTi LDO catalyst in Figure 4g demonstrates a lower overpotential than CPM, its synthesis involves expensive materials such as RuCl_3_ (£463 per 10 g) and Ni foam, making overall production significantly more costly. In contrast, CPM is far more cost‐effective with a total material cost of ≈ £2–£5 per gram. This indicates that the CPM catalyst could be a promising alternative to expensive catalysts for reducing the potential gap between the discharge and charge processes. Li‒CO_2_ batteries based on 40:50 and 20:70 cathodes also produced lower overpotentials than that of Super P cathode (1.61 V), indicating the high capability of the CPM catalyst to lower the charge potential and accelerate the reversible reaction. Compared to Super P, the 80:10 cathode displayed a remarkable reduction in overpotential from 1.61 to 0.67 V.

Accordingly, this overpotential reduction likely extended the cycle life of the battery, as verified by the long‐term stability of Li‒CO_2_ batteries at 50 mA g^−1^ within the cut‐off capacity of 500 mAh g^−1^. As shown in Figure 4d, the Li‒CO_2_ battery with the 80:10 cathode displayed a long stability of 107 cycles, ≈5.9 times longer than the cycle life of Super P (Figure S6b, Supporting Information). The batteries with 20:70 and 40:50 cathodes ran for 37 cycles (Figure S6c, Supporting Information) and 76 cycles (Figure S6d, Supporting Information), respectively. Additionally, the 90:0 cathode operated for 41 cycles (Figure S6e, Supporting Information), lasting longer than the 20:70 and Super P cathodes but falling short of the 80:10 and 40:50 cathodes. This shows that while CPM alone enhances cycling stability, an optimal catalyst‐to‐Super P ratio is essential for further improving durability and overall battery performance. The longer cycle life of the CPM‐based batteries suggests the superior electrocatalytic performance of the CPM catalyst in enhancing the reversibility of Li‒CO_2_ reactions (Figure 4e). Even with 20% and 40% of the CPM catalyst, the cyclability increased by nearly 2 and 4.2 times, respectively, compared to the Super P cathode. Thanks to the high stability of the CPM catalyst, the 80:10 cathode consistently kept the charge potential of the Li‒CO_2_ battery below 4 V throughout cycling, maintaining it between 3.5 and 3.7 V during the first 30 cycles as shown in Figure 4e. Overall, the battery with the 80:10 cathode could cycle for more than 2100 h, whereas the Super P‐based battery lasted only 360 h (Figure 4f), highlighting the superb long‐term cyclability facilitated by the CPM catalyst.

The high electrocatalytic performance of CPM can be attributed to the uniform distribution of active sites and the mesoporosity of the catalyst. These structural features not only enhance CO_2_ diffusion and ion transport but also offer sufficient pore volume for Li_2_CO_3_ deposition/decomposition during cycling. Consequently, this improves catalytic reaction kinetics, reduces polarization during cycling, and leads to enhanced charge–discharge capacities.

The 80:10 CPM cathode demonstrated good cycle life and stability compared to reported catalysts. For instance, the RuO_2_@CNT catalyst^[^ ^54^ ^]^ operated for only 55 cycles, whereas the 80:10 cathode achieved nearly double that under similar conditions (Figure 4d). Additionally, the 80:10 cathode sustained operation for over 2100 h, far exceeding the lifetimes of some noble metal‐based catalysts such as Ru@Super P (cycled for 1600 h),^[^ ^20^ ^]^ IrO_2_@CNT (operated for 992 h),^[^ ^57^ ^]^ and RuNi@MWCNT (lasted just 415 h)^[^ ^53^ ^]^ (Table S1, Supporting Information). These comparisons underscore the long‐term stability and robustness of the cost‐effective CPM catalyst, making it a competitive alternative to expensive noble metal catalysts. It is noteworthy that increasing the amount of CPM catalyst enhanced the electrochemical performance and extended the cycle life of the Li‒CO_2_ batteries. However, the 90:0 cathode did not surpass the performance of the 80:10 cathode, highlighting the importance of a balanced ratio of CPM and Super P for achieving optimal electrochemical performance. This is most likely due to CPM being too electronically insulating without the addition of conductive Super P, which limits efficient charge transfer during battery operation. Among the tested cathodes, the 80:10 composition demonstrated the highest discharge capacity, lowest overpotential, and longest cycle life, making it the optimal choice for improving battery efficiency and a promising candidate catalyst for future Li‒CO_2_ batteries.

### 2.3 Investigation of CPM Cathode After Discharge–Charge Process

**Figure 5:**
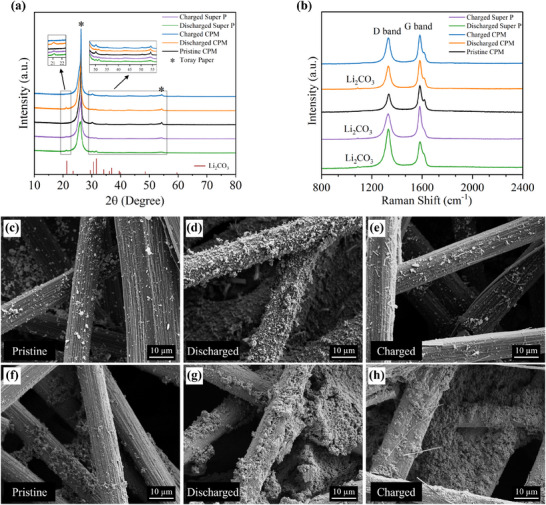
a) PXRD patterns and b) Raman spectra of 80:10 CPM cathodes at pristine, discharged, and charged stages, along with Super P cathodes at discharged and charged stages. SEM images of c) pristine, d) discharged, and e) charged 80:10 CPM cathodes at an acceleration voltage of 5 kV. SEM images of Super P cathodes at f) pristine, g) discharged, and h) charged stages at 3 kV acceleration voltage.

To further study the stability, reversibility, and electrochemical reaction mechanism of the best‐performing CPM electrode (80:10) in the Li‒CO_2_ battery, the cathodes were investigated using post‐mortem ex‐situ characterization in states of discharge and charge. **Figure** **5**a displays the PXRD profile of the CPM cathode at different stages of pristine, discharged and charged, as well as the Super P cathodes at the discharged and charged stages. Compared to the pristine CPM cathode, the PXRD pattern of the CPM cathode after full discharge displays new peaks at 21.24, 31.71, 33.92, and 36.82°, which are assigned to (1 1 0), (0 0 2), (1 1 2), and (3 1 1) crystal planes of discharge product Li_2_CO_3_, respectively (JCPDS 09–0359). After recharge, all the diffraction peaks corresponding to the Li_2_CO_3_ disappeared, revealing the successful largely reversible reaction (CE of 97.3%) between crystalline Li_2_CO_3_ and carbon promoted by the 80:10 CPM cathode during battery charging. This also aligns with the high CE achieved by the Li‒CO_2_ battery based on the CPM cathode. In contrast, the PXRD pattern of the Super P cathode after full recharge still exhibits the characteristic peaks of Li_2_CO_3_, confirming the incomplete decomposition of discharge products and poor reversibility, as indicated by the low CE of 13.8%.

XRD results were further verified by ex‐situ Raman analysis as seen in Figure 5b. Two characteristic peaks of pristine CPM cathode were identified at 1329 and 1582 cm^−1^, which are assigned to the D band (disorder band) and G band (graphitic band) of carbon, respectively.^[^ ^64^ ^]^ The discharged CPM cathode showed a new peak at ≈1089 cm^−1^ attributed to the symmetric C−O stretching vibrational mode of the carbonate group in Li_2_CO_3_, exhibiting the formation of Li_2_CO_3_ after discharge.^[^ ^65^ ^]^ This observed peak vanished after the full recharge because of the high capability of the CPM catalyst to facilitate the Li_2_CO_3_ decomposition. However, the Li_2_CO_3_ peak remained visible after a full recharge of the Super P cathode, suggesting that the discharge products were not completely decomposed.

The observations from the ex‐situ SEM characterization also revealed the formation and oxidation of discharge products on the CPM electrode (Figure 5c‒e), validating the XRD and Raman findings. Figure 5d clearly illustrated that Li_2_CO_3_ particles fully covered the surface of the CPM cathode after discharge, while the electrode morphology returned to close to its original form (Figure 5c) after recharging. The Li_2_CO_3_ particles were successfully decomposed (Figure 5e) due to the superior electrocatalytic activity of the CPM catalyst, which effectively accelerated the reversible CO_2_ conversion. In comparison, the SEM images of the Super P cathode at pristine, discharged, and charged stages (Figure 5f‒h) showed that Li_2_CO_3_ particles largely remained after recharging. Instead of returning to its original pristine morphology, the electrode maintained a structure similar to the discharged state, highlighting the limited catalytic activity of the Super P cathode in facilitating reversible CER.

**Figure 6:**
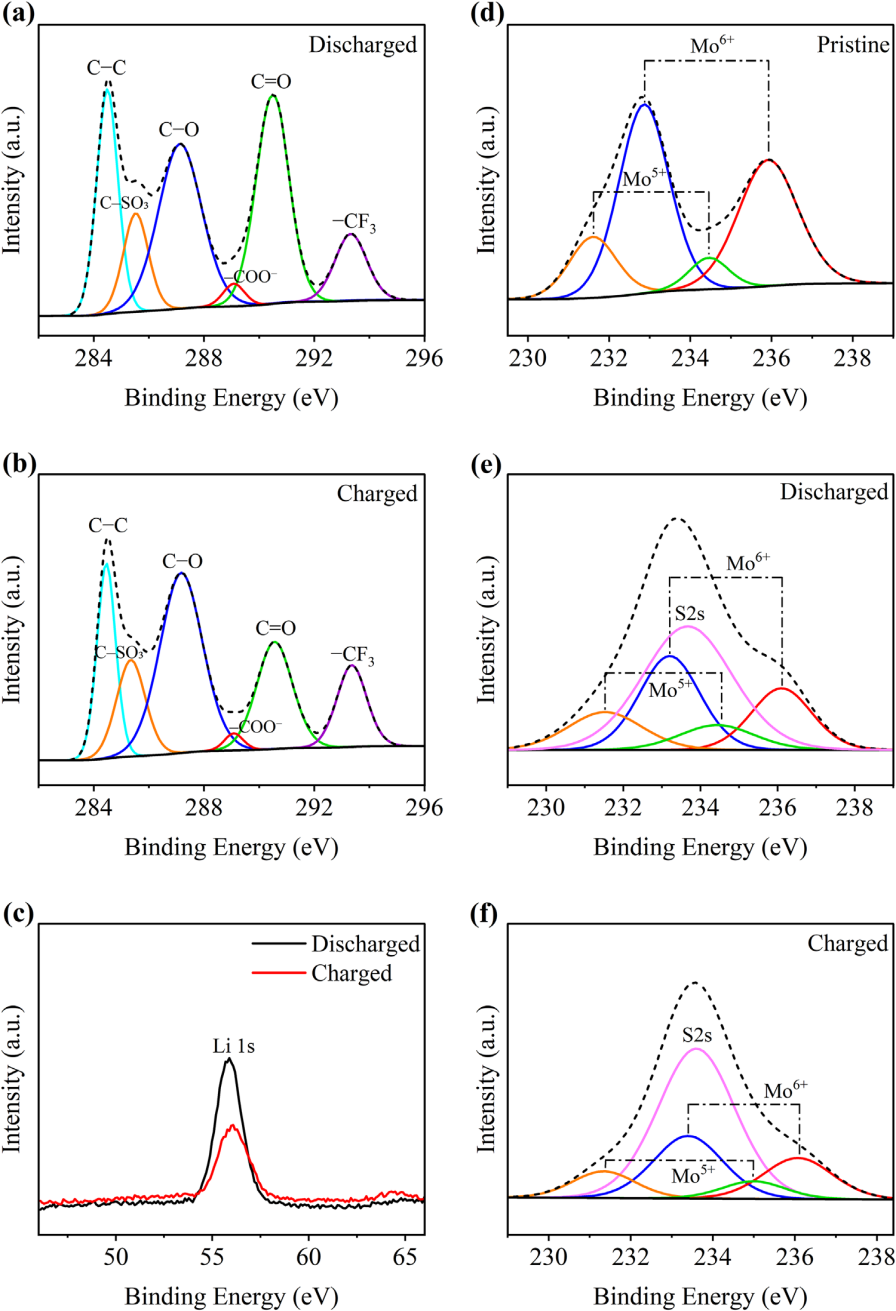
High‐resolution XPS spectra of the 80:10 CPM cathode: a) C 1s (discharged), b) C 1s (charged), c) Li 1s after discharge and charge, d) Mo 3d (pristine), e) Mo 3d (discharged), and f) Mo 3d (charged).

Ex‐situ XPS characterization was subsequently utilized to analyze the changes in the surface states of the CPM cathodes after discharge and recharge (**Figure** **6**). As shown in Figure 6a, the high‐resolution XPS spectra of C 1s for the discharged CPM cathode revealed an intense peak at 290.6 eV, corresponding to carbonate (CO_3_^‒^) group in the discharge product Li_2_CO_3_.^[^ ^66^ ^]^ The carbonate peak was significantly reduced after recharge (Figure 6b), confirming the Li_2_CO_3_ decomposition via the predominantly reversible reaction in the Li‒CO_2_ battery. However, the carbonate peak was not eliminated after charging, which indicates that a small amount of discharge products could not be decomposed, explaining the battery's CE of 97.3%. The C 1s spectrum also identified the characteristic peaks associated with C−O of tetraglyme electrolyte, C−SO_3_ (sulfonate group of LiTFSI), and −CF_3_ within LiTFSI.^[^ ^53^, ^67^, ^68^ ^]^ Meanwhile, a similar variation was observed for Li 1s peak at 55.8 eV (Figure 6c), further substantiating the formation and decomposition of discharge products based on the reversible reaction catalyzed by the high‐performance CPM catalyst in the Li‒CO_2_ battery.

Intriguingly, the XPS spectra of the Mo element after discharge and recharge of the CPM cathode offer valuable insights into the effective participation of the catalyst in facilitating the CRR and CER processes. Figure 6d shows detection of two oxidation states for Mo (+6 and +5) on the surface of the pristine CPM@Super P cathode. After discharge, the share of the Mo^5+^ state steeply declined, whereas that of the Mo^6+^ increased (Figure 6e), explaining the oxidation of Mo in the lower valence state (Mo^5+^) to Mo in the higher valence state (Mo^6+^). This elucidates how CPM acts as an excellent electron donor, effectively catalyzing the CO_2_ adsorption and reduction in Li‒CO_2_ batteries.^[^ ^69^ ^]^ Upon recharging, the delocalized electrons migrated back to Mo, which restored the proportions of Mo^5+^ to their initial state, thereby underscoring the largely reversible CO_2_ conversion driven by the Mo active sites of the CPM catalyst (Figure 6f). Additionally, a S 2s sulphonate peak (≈233.8 eV) convoluted with the Mo 3d peak was observed in Figure 6e,f, originating from the sulfonate group of LiTFSI present in the residual electrolyte on the cathode surface. These results, which are in good agreement with the previous observations gained by the ex‐situ XRD, Raman, SEM, and electrochemical measurements, demonstrate that the CPM cathode promotes reversible CRR/CER kinetics and lowers the charge overpotential.

## 3 Computational Investigation of the Detailed Mechanism of Catalysis

After identifying the discharge‐charge products and assessing the reversibility, the catalytic mechanism of CPM was investigated using density functional theory (DFT) calculations. First, the surface energy (γ) of the low‐Miller index surfaces of the CPM catalyst─(100), (110), and (111)─was estimated using Equation (1). The calculated surface energy values were 3.81 J m^−2^ for (100), 1.43 J m^−2^ for (110), and 1.00 J m^−2^ for (111), indicating that (111) is the most stable facet.

A screening of different adsorption configurations of Li, CO_2_, C and Li_2_CO_3_ was made on the CPM(100) surface, as this surface exhibits greater exposure to various surface interactions, including Cs‒Cs bridge sites, Mo‒Cs bridge sites with high and low oxygen concentrations, and P‒Mo sites (Figure S7a,b and Table S2, Supporting Information). Figure S7a (Supporting Information) displays that Li, with a more negative adsorption energy, shows significantly stronger adsorption on the CPM(100) surface compared to CO_2_. This observation suggests that reaction pathways initiated by CO_2_ adsorption (* + CO_2_ → *CO_2_, where * denotes the basal plane of CPM(100)) are less likely to occur.

**Figure 7:**
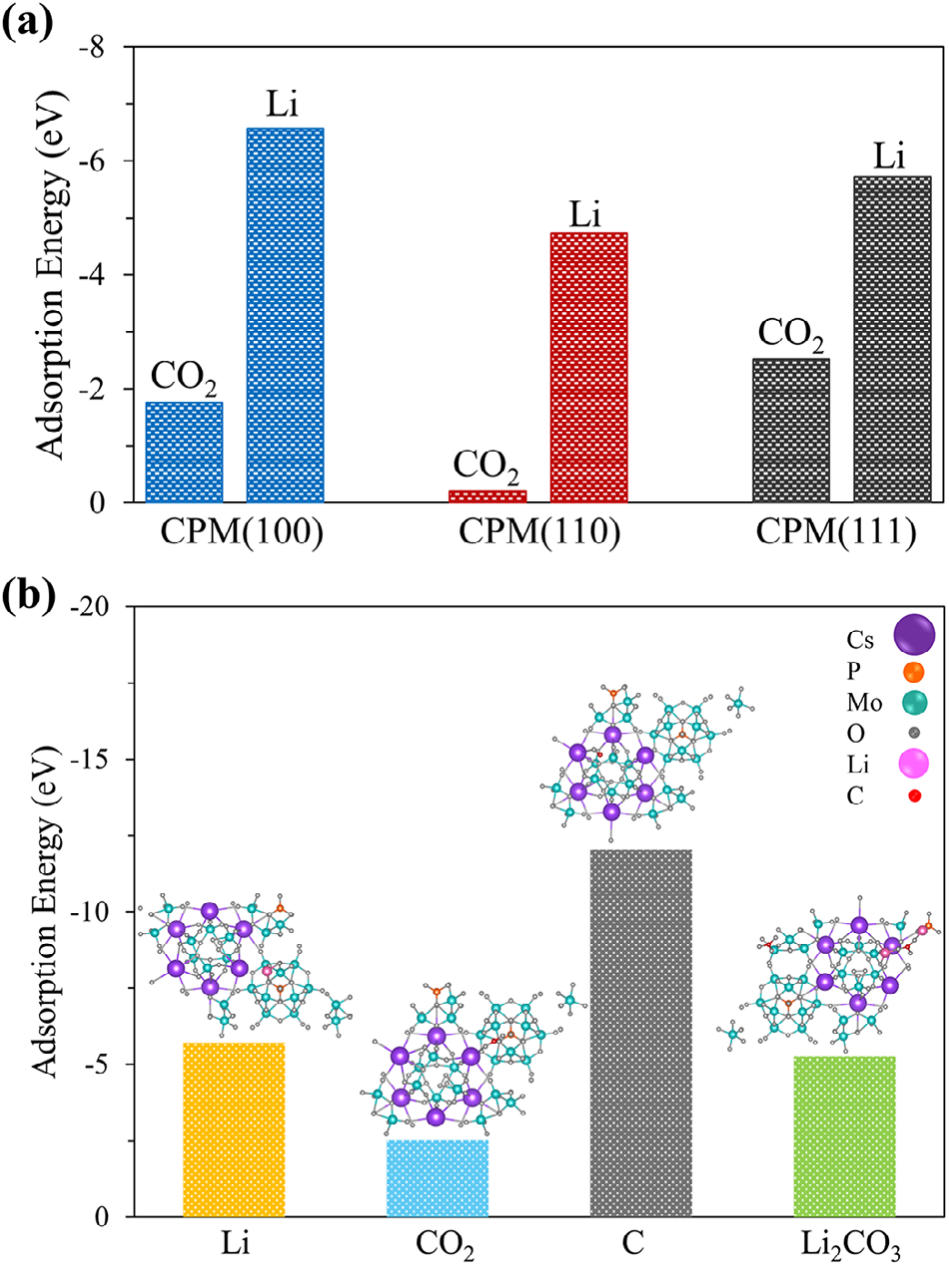
a) Adsorption energy of Li and CO_2_ on the (100), (110), and (111) surfaces of the CPM catalyst. The bridge Cs‒Mo adsorption configuration was considered for all surfaces, as it was identified as the most favorable adsorption site. b) Adsorption energies for Li, CO_2_, C and Li_2_CO_3_ on the CPM(111) surface. Insets display the top view of Li, CO_2_, C and Li_2_CO_3_ adsorption configurations on the CPM(111) surface.

In addition, the adsorption of Li and CO_2_ was investigated on the CPM(110) and CPM(111) surfaces at Cs‒Mo bridge sites, identified as the most favorable adsorption sites. **Figure** **7**a exhibits that the adsorption energy of Li is considerably higher than that of CO_2_ on all observed facets, demonstrating that the mechanism initiates with Li rather than CO_2_, regardless of the most exposed facet.

Finally, the favorable adsorption configurations of species were determined on the most stable surface, CPM(111), and reported in Figure 7b. The side view (Figure S7c, Supporting Information) and top view (Figure 7b) of Li, CO_2_, C and Li_2_CO_3_ adsorption configurations on the CPM(111) surface reveal that the most favorable adsorption configurations predominantly occur at Mo–O and Cs–O sites. Notably, the most favorable adsorption mode of carbon atoms is amidst oxygen atoms, specifically over the Cs–O sites, assuming the CO_3_ trigonal planar configuration.

Given that the adsorption energy of Li_2_CO_3_ (−5.26 eV) is lower than that of C (−12.04 eV) on the CPM(111) surface, three potential reaction pathways were proposed for the formation of *C and Li_2_CO_3_ based on insights from recent literature.^[^ ^70^, ^71^, ^72^, ^73^ ^]^ Wang et al.^[^ ^74^ ^]^ demonstrated that the most feasible route for the formation of Li_2_CO_3_ and C on the Pt(111) surface is as follows:

(1)

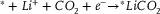



(2)

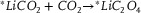



(3)

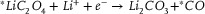



(4)

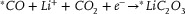



(5)

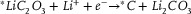




Given the similar adsorption energy trends observed in the present study, it is reasonable to propose that this pathway may also be the most viable pathway for forming Li_2_CO_3_ and C on the CPM(111) surface. Compared to the Pt catalyst reported in a previous study,^[^ ^74^ ^]^ CPM demonstrates significantly stronger adsorption of reactants, including Li (−5.72 eV) and CO_2_ (‐2.53 eV), enabling efficient intermediate formation and enhanced reaction kinetics. In contrast, Pt exhibits weaker adsorption energies for these reactants (−3.31 eV for Li and −0.23 eV for CO_2_), which may constrain its catalytic activity. Nonetheless, Pt's moderate adsorption of Li_2_CO_3_ (−2.48 eV) and carbon (−8.7 eV), compared to CPM, suggests better resistance to surface passivation over time. Despite this, CPM's strong affinity for reactants highlights its potential as a highly effective catalyst for applications prioritizing reaction efficiency and performance.

Notably, Figure S7a (Supporting Information) suggests that the trends of adsorption energy values for Li, CO_2_, Li_2_CO_3_, and C on CPM(100) are similar to those on the CPM(111) surface (Figure 7b), confirming that the reaction mechanism remains consistent across different catalyst facets. Moreover, Figure S7d (Supporting Information) illustrates top and side views of Li and CO_2_ adsorption configurations on the CPM(110) surface.

## 4 Conclusion

In this study, CPM was successfully employed as a bifunctional electrocatalyst to facilitate both the CRR and CER processes in Li‒CO_2_ batteries for the first time. An electrode coated with an optimal catalyst:Super P ratio (80:10) lowered the potential of the reversible charge reaction of Li‒CO_2_ batteries to 3.5 V, exhibiting an exceptional overpotential of only 0.67 V, which is much lower than that with most previously reported catalysts. Additionally, the battery could also deliver a high discharge capacity of 15 440 mAh g^−1^ and maintain stable reversibility over 107 cycles. The unique structure of oxo‐bridged Mo atoms in the highest oxidation state (+6) provided abundant active sites to promote CO_2_ adsorption and release during battery cycling. This catalysis was further examined through ex‐situ characterization, confirming the superior catalytic capability of the CPM catalyst in accelerating the nearly reversible formation and decomposition of discharge products. This research can provide new perspectives on the development and application of heteropolymolybdate catalysts for reversible Li‒CO_2_ batteries, not only because of their excellent electrocatalytic performance in reducing the reversible charge potential but also due to their low cost, ease of synthesis, robust stability, and environmentally benign nature. Future research is recommended to explore various heteropolymolybdate catalysts, refine the catalyst‐electrode interface for improved charge transfer, and examine how electrolyte composition affects the catalyst's performance in Li–CO_2_ batteries.

## 5 Experimental Section

### Materials

Caesium nitrate (CsNO_3_), isopropyl alcohol (IPA), polyvinylidene fluoride (PVDF), N‐Methyl‐2‐pyrrolidone (NMP), glass fiber (Whatman, Grade GF/F), lithium triflate (LiCF_3_SO_3_), tetraglyme (tetraethylene glycol dimethyl ether, TEGDME), and molecular sieves (40 Å) were purchased from Sigma–Aldrich (UK). Phosphomolybdic acid (PMA, H_3_PMo_12_O_40_) hydrate and Triton‐X‐100 (C_16_H_26_O_2_) were purchased from Thermo Fisher Scientific (UK). Carbon Super P (MTI, USA), Toray carbon paper (TGP‐H060, thickness 0.19 mm) and Li discs (99.9%, thickness 0.6 mm) purchased from Soion Technology Ltd (UK) were used.

### Synthesis of CPM Nanocomposite

PMA and CsNO_3_ at a molar ratio of 1:3 with seven drops of Triton‐X‐100 as a surfactant were thoroughly ground in an agate mortar until a uniform yellow powder was formed (Figure 1). The prepared sample was then sonicated in isopropyl alcohol for 20 min and separated by centrifugation. This process was repeated five times to ensure the removal of all impurities. Finally, the wet CPM particles were dried overnight at 50 °C in a vacuum oven.

### Material Characterization

PXRD was used to identify the crystalline structure and chemical composition of the synthesized samples using the PANalytical X'Pert diffractometer (Royston, UK) with Ni‐filtered Cu‐K𝛼 radiation (λ = 1.54059 Å) at a voltage of 40 kV and current of 30 mA. PXRD patterns were collected using a PIXcel‐1D detector over a scan range of 2θ = 10°–70°. PXRD pattern was indexed using the TOPAS program.^[^ ^39^, ^40^ ^]^ Details of the Pawley fit^[^ ^75^ ^]^ and Rietveld refinement^[^ ^42^ ^]^ can be found in the Supporting Information. The chemical structure and functional groups of the catalyst were investigated using FTIR spectrometry on a Spectrum Two FT‐IR Spectrometer (PerkinElmer, USA) over the spectral range of 4000–400 cm^−1^ and a resolution of 4 cm^−1^. Raman spectroscopy was also used to identify the chemical composition and specific molecular bonds of the as‐synthesized CPM catalyst using an inVia confocal Raman microscope (Renishaw, UK) with a 532 nm laser excited from a helium‐neon source. Given the thermal sensitivity of the CPM catalyst, it was crucial to set a low laser power (2.5 mW) to prevent sample decomposition. Raman spectra were measured over the scanning range of 100–1200 cm^−1^ with 10 s exposure and 10 repetitions. XPS spectra were recorded on a Thermo Fisher Scientific K‐Alpha+ spectrometer (East Grinstead, UK) equipped with monochromated Al Kα radiation at 12 kV to qualitatively analyze surface chemistry and elemental composition.

The amount of bound water in the CPM composite was determined by TG using the Discovery TGA 550 (TA Instruments, UK). Under a nitrogen atmosphere, the sample was heated between 30 and 400 °C at a heating rate of 10 °C min^−1^. The mass loss determined by TG was converted to the stoichiometric number of water molecules in the sample. Nitrogen adsorption‐desorption analysis was used to study the pore size and specific surface area of the synthesized catalyst using the Belsorp mini II (Japan). Prior to analysis, the sample was outgassed under N_2_ at 50 °C for a week. Using N_2_ adsorption‐desorption isotherms, the pore volume and distribution of pore sizes were calculated through BET and BJH methods, respectively.

SEM and EDS were performed with the TF APREO 2 SEM (ThermoScientific) to observe the surface morphology and elemental composition of the samples. The acceleration voltage of the EDS analysis was 10 kV. The surface morphology and atomic spacing were further studied by TEM, SAED, and HAADF‐STEM using a Talos F200i TEM (ThermoScientific) at an acceleration voltage of 200 kV.

### Preparation of Cathode Electrodes

80% of CPM catalyst, 10% of carbon Super P, and 10% of PVDF binder were mixed in NMP and then ball‐milled to achieve a uniform black slurry. Subsequently, the slurry was uniformly coated on the surface of Toray carbon paper, which was precut into 14 mm diameter disks. The CPM‐coated carbon paper disks were dried in a vacuum oven at 50 °C overnight to be employed as cathode electrodes (named 80:10 cathodes) in Li–CO_2_ batteries. The mass loading of the active material was ≈0.1–0.2 mg cm^−2^. For comparison, the same procedure was used to fabricate two additional CPM‐based cathodes with two different mass ratios of CPM:Super P:binder = 40:50:10 and 20:70:10 (referred to as 40:50 and 20:70 cathodes, respectively). Additionally, CPM cathodes without Super P (designated as 90:0 cathodes) were prepared, along with Super P cathodes (without CPM catalyst).

### Battery Assembly and Electrochemical Measurements

Meshed CR2032‐type coin cells (SOION Technology Ltd, UK) with several holes on the cathode cap to expose the electrode to CO_2_ were assembled in an Ar‐filled glove box (O_2_ < 0.5 ppm, H_2_O < 0.5 ppm). The fabricated CPM electrodes (80:10, 40:50, and 20:70 electrodes) and Super P electrode were directly assembled as cathodes of Li–CO_2_ batteries. Li metal chip (14 mm in diameter) and glass fiber (16 mm in diameter) were used as the anode electrode and separator, respectively. The electrolyte was prepared by dissolving LiCF_3_SO_3_ salt in TEGDME solvent at a molar ratio of 1:4 in the Ar‐filled glove box. Prior to electrolyte preparation, the Li salt underwent vacuum drying at 100 °C for 24 h while the TEGDME solvent was dried using molecular sieves for at least three days. As shown in Figure S8a (Supporting Information), Li–CO_2_ batteries were assembled by stacking cell components as follows: anode case, spring, spacer, Li anode, glass fiber separator soaked in the electrolyte solution, prepared cathode electrode, and cathode case. Afterwards, the assembled coin cells were sealed in a locally designed glass assembly (Figure S8b, Supporting Information) in the glove box and were then purged with pure CO_2_ gas for 15 min outside the glove box.

After stabilizing the batteries, constant current discharge‐charge measurements were performed at a voltage range of 2.0–4.4 V using a Neware BTS 8.0 battery tester (China). All specific capacities and current densities of cathode electrodes were calculated using the mass of active material coated onto the carbon paper. CV analysis was recorded within the potential range of 2.0–4.4 V at a scan rate of 0.5 mV s^−1^ using a Gamry interface 1010E potentiostat.

The discharged and charged cathode electrodes were taken out of the cells for post‐mortem characterization by PXRD, Raman, SEM, and XPS. Raman spectroscopy was conducted ex‐situ to characterize the surface composition of discharged and charged cathode electrodes at the 633 nm excitation line and constant power of 10 mW (helium‐neon source). The data were recorded over the scanning range of 800−2400 cm^−1^ with 10 s exposure and 10 repetitions.

### Computational Method

DFT calculations were performed based on the periodic boundary conditions formalism, as implemented in the Vienna ab initio Simulation Package (VASP).^[^ ^76^, ^77^, ^78^, ^79^ ^]^ A cut‐off energy of 650 eV and a 3×3×1 Γ‐centered Monkhorst‐Pack *k*‐points mesh were employed. The Perdew‐Burke‐Ernzerhof (PBE) functional and the DFT‐D3 method with Becke‐Jonson (BJ) damping van der Waals corrections were used.^[^ ^80^, ^81^ ^]^ The energy tolerance for the self‐consistent field calculations was set to 1×10^−6^ eV, and the forces were considered converged when all values were smaller than 0.05 eV Å^−1^.

The surface energy (γ) values were estimated using Equation (6).

(6) $$\gamma =\frac{E_{slab}-NE_{bulk}}{2A}$$

where *E_slab_* is the DFT energy of the surface, *E_bulk_* is the energy of the bulk catalyst and N is the number of atoms in the surface. For adsorption investigations, the slabs were created to ensure that periodic images of the adsorbate were separated by at least 10 Å. Furthermore, a vacuum region of 20 Å was included in the non‐periodic axis. The adsorption energy (*E_ads_*) was calculated using Equation (7).

(7) $$E_{ads}=E_{adsorbate-surf}\;-E_{surf}-E_{adsorbate}$$

where *E_adsorbate‒surf_* is the energy of the adsorbed systems, *E_surf_* is the energy of the bare surface and *E_adsorbate_* is the energy of the isolated molecule/atom in a vacuum box of 20 Å.

## Conflict of Interest

The authors declare no conflict of interest.

## Supporting information



Supporting Information

## Data Availability

The data that support the findings of this study are available from the corresponding author upon reasonable request.
