# Supplementary material for: Ultralow Overpotential in Rechargeable Li–CO2 Batteries Enabled by Caesium Phosphomolybdate as an Effective Redox Catalyst
Source: Adv Sci (Weinh). 2025 Apr 30;12(27):2502553. doi: 10.1002/advs.202502553 (PMC12279220; doi:10.1002/advs.202502553)
Supplement: Supplementary file 1 — Supporting Information [file ADVS-12-2502553-s001.pdf]

ADVANCED SCIENCE
Open Access

Supporting Information

for *Adv. Sci.*, DOI 10.1002/advs.202502553

Ultralow Overpotential in Rechargeable Li–CO_2 Batteries Enabled by Caesium Phosphomolybdate as an Effective Redox Catalyst

Mahsa Masoudi, Neubi F. Xavier Jr, James Wright, Thomas M Roseveare, Steven Hinder, Vlad Stolojan, Qiong Cai, Robert C. T. Slade, Daniel Commandeur and Siddharth Gadkari**

Supporting Information

Ultralow Overpotential in Rechargeable Li–CO_2 Batteries Enabled by Caesium Phosphomolybdate as an Effective Redox Catalyst

Mahsa Masoudi[a], Neubi Xavier[a], James Wright[a], Thomas M Roseveare[b], Steven Hinder[c], Vlad Stolojan[d], Qiong Cai[a], Robert C. T. Slade[a], Daniel Commandeur[a], Siddharth Gadkari[a]**

[a] School of Chemistry and Chemical Engineering, Faculty of Engineering and Physical Sciences, University of Surrey, Guildford GU2 7XH, United Kingdom.

[b] Department of Chemistry, University of Sheffield, Brook Hill, Sheffield S3 7HF, United Kingdom.

[c] School of Mechanical Engineering Sciences, Faculty of Engineering and Physical Sciences, University of Surrey, Guildford GU2 7XH, United Kingdom.

[d] Advanced Technology Institute, School of Computer Science and Electronic Engineering, Faculty of Engineering and Physical Sciences, University of Surrey, Guildford GU2 7XH, United Kingdom.

*Corresponding authors:

Dr. Siddharth Gadkari: s.gadkari@surrey.ac.uk

Dr. Daniel Commandeur: d.commandeur@surrey.ac.uk

Rietveld Refinement on Keggin Structure

The PXRD pattern was indexed using the TOPAS program,[1] in conjunction with jEdit, wherein a cubic primitive unit cell similar to the reported structure was observed.[2] At least two weak reflections at 19.9° and 28.3° were observed which did not match this cell and appeared to be due to the presence of a small impurity from the $CsNO_3$ starting material. The observed primitive cubic unit cell, as well as that of $CsNO_3$ (Data retrieved from the Materials

Project for $CsNO_3$ (mp-561851) from database version v2024.12.18.)[3] were used as the starting point for a mixed-phase Pawley refinement,[4] employing 332 parameters (7 background, 1 zero error, 9 profile, 3 unit cell, 312 reflections), resulting in indices of fit Rwp = 2.051, Rwp' = 10.62. [**$Cs_3PMo_{12}O_{40}$** (Cubic, *Pn-3m*): a = 11.7766 (4) Å; **$CsNO_3$** (Hexagonal, *$P3_1$*) a = 10.9143 (6) Å, c = 7.7287 (5) Å].

The pattern shown in **Figure S1** was then used for Pawley refinement to determine the structure of $Cs_3PMo_{12}O_{40}$. A structural model of the $[PMo_{12}O_{40}]^{3-}$ cluster was adapted from the published crystal structure of $H_3PW_{12}O_{40}$,[5] which crystallized in the same space group and a similar cubic unit cell. Waters of crystallization/hydroxonium ions from the published $H_3PW_{12}O_{40}$ structure were replaced at fractional coordinates [0.75, 0.25, 0.25] with Cs atoms. The published structure of $CsNO_3$ was used as the structural model for this phase in a mixed-phase Rietveld refinement[6] of the pattern. The unit cells derived from the Pawley refinement outlined above were the starting point for this analysis. Mixed-phase Rietveld refinement employed 25 parameters (7 background, 1 zero error, 9 profile, 3 cell, 2 scale, 3 thermal parameters – one for the $[PMo_{12}O_{40}]^{3-}$ cluster, one for Cs atoms and one for $CsNO_3$ structure), resulting in indices of fit Rwp = 2.852, Rwp' = 14.67 (**Figure 2a**).

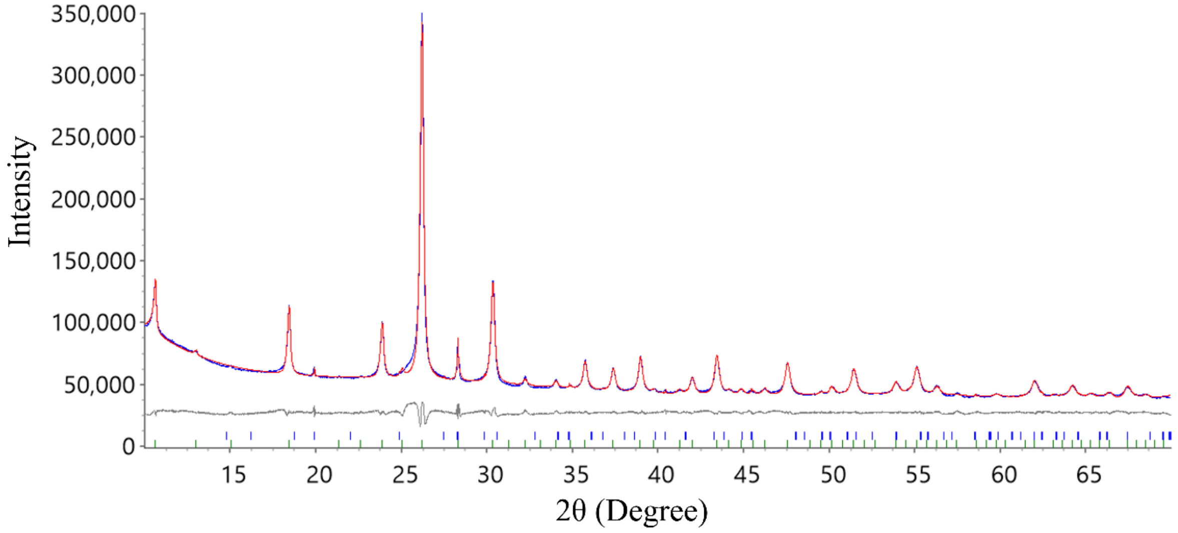

Figure S1. Observed (blue) and calculated (red) profiles and difference plot ($I_{obs} - I_{calc}$) (grey) of the mixed-phase Pawley refinement. (2θ=10–70°, d_{min}= 1.34 Å).

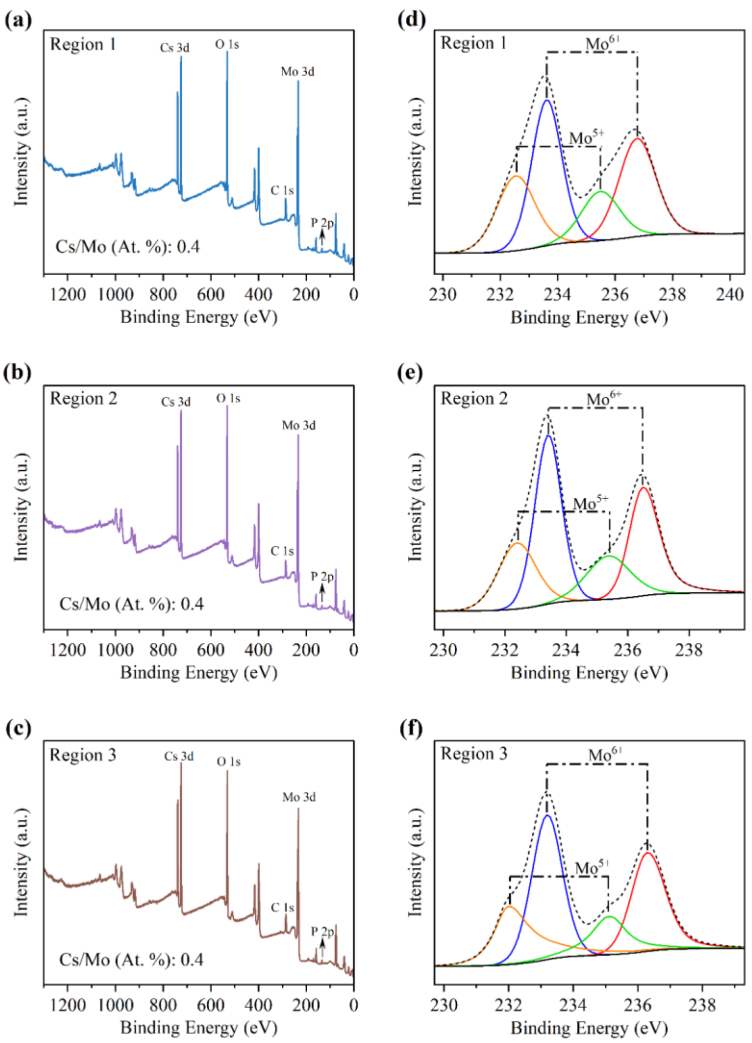

Figure S2. XPS analysis of CPM catalyst in three different regions: (a–c) survey spectrum, and (d–f) high-resolution XPS spectra of Mo 3d.

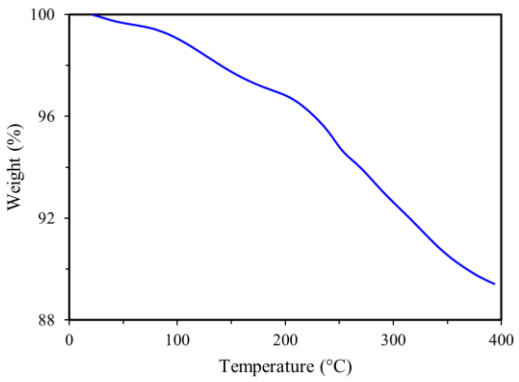

Figure S3. (a) TG of the synthesized CPM catalyst.

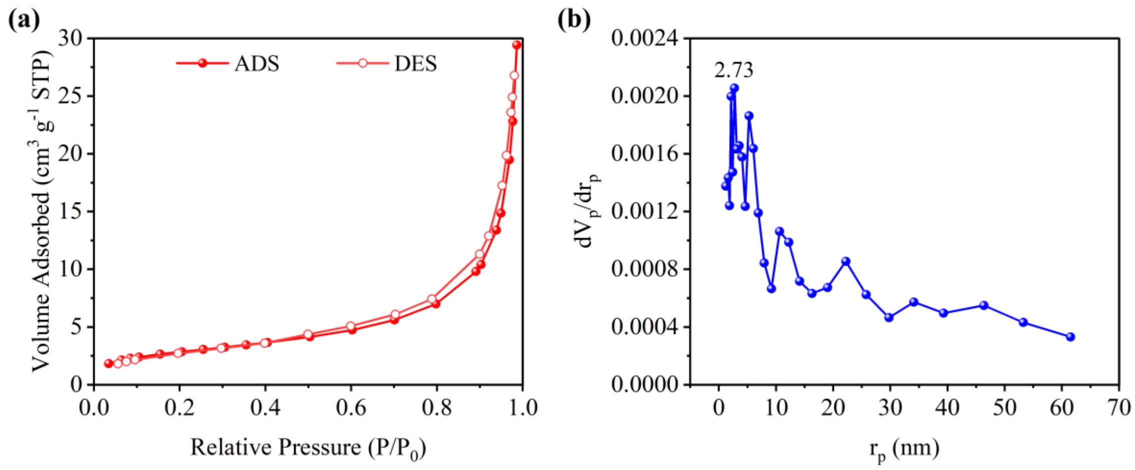

Figure S4. (a) N_2 adsorption-desorption isotherm and (b) BJH pore size distribution of the CPM catalyst.

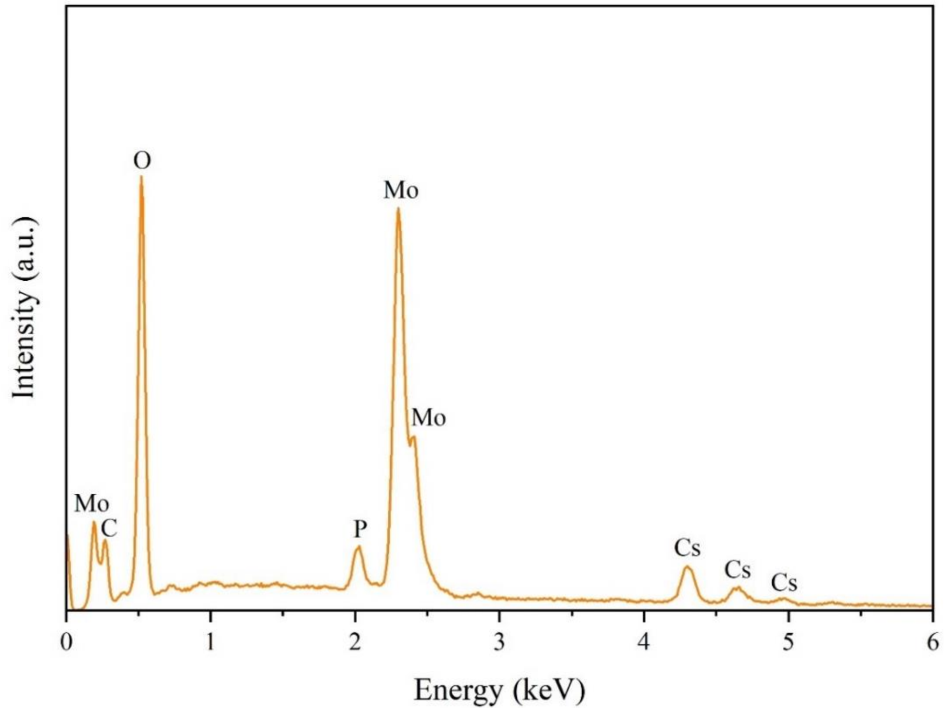

Figure S5. EDS spectrum of the CPM nanocomposite.

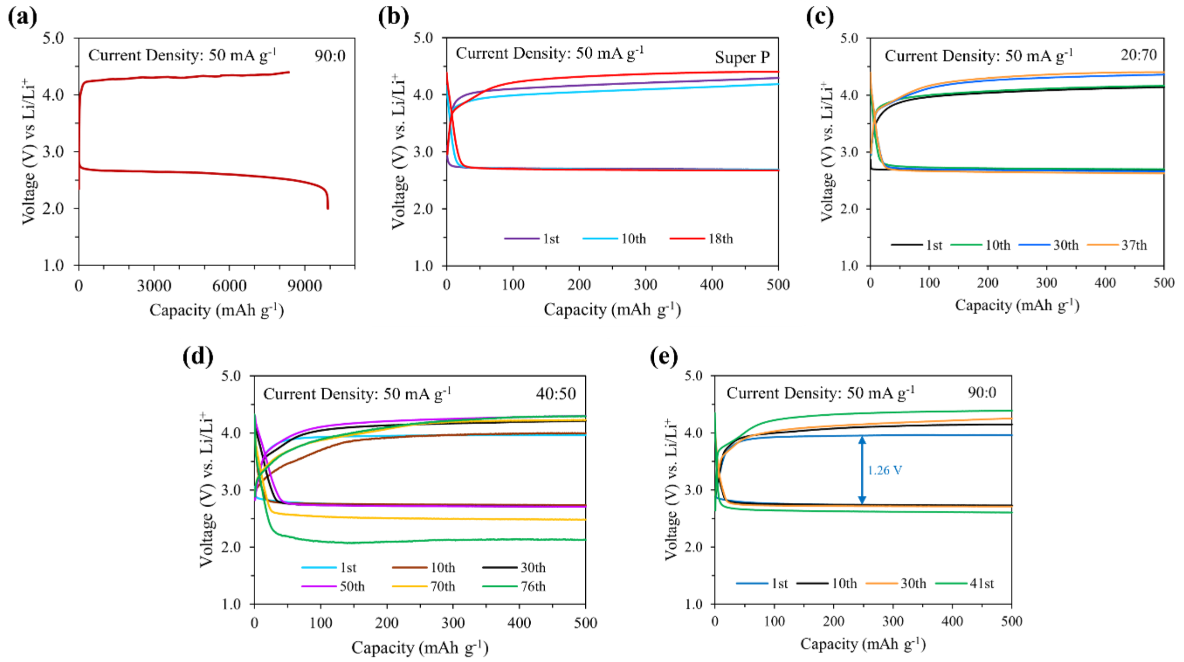

Figure S6. (a) Full GCD of the Li–CO_2 battery with 90:0 cathode at the current density of 50 mA g^{-1}. Cycling performance of Li–CO_2 batteries with (b) Super P, (c) 20:70, (d) 40:50, and (e) 90:0 cathodes at 50 mA g^{-1} and limited capacity of 500 mAh g^{-1}.

Table S1. Performance comparison of different cathode catalysts in Li–CO_2 batteries reported in the literature.

Catalyst	Discharge Capacity [mAh g^{-1}] /Applied Current [mA g^{-1}]	C.P. / O.P. [V]	C.E. [%]	Cycle Performance (Current Density [mA g^{-1}] /Cut-off Capacity [mAh g^{-1}])	Ref.
CPM@Super P	15438 /50	3.5/0.67	97.3	107 cycles (50 /500)	This work
Ru@Super P	8229 /100	4.25/1.0	86.2	80 cycles (100 /1000)	[7]
RuO_2@CNT	2187 /50	3.9/1.3	93	55 cycles (50 /500)	[8]
RuNi@MWCNT	15165 /50	3.8/1.15	97.4	83 cycles (200 /500)	[9]
RuCo@CNT	8057 /100	3.74/0.94	N/A	N/A	[10]
IrO_2@CNT	3347 /100	4.24/1.63	N/A	124 cycles (100 /400)	[11]
IrRu@N-CNT	6228 /100	-/1.1	100	7660 h (100 /500)	[12]
NiO@CNT	9000 /50	4.0/1.2	97.8	42 cycles (50 /1000)	[13]
Co–NiO@CNT	5871 /100	3.94/1.27	92.91	50 cycles (100 /500)	[14]
Mn_2O_3@KB	9434 /50	-/1.4	N/A	50 cycles (50 /1000)	[15]
RuCu@CNF	15753 /300	3.7/0.9	99.3	110 cycles (500 /1000)	[16]
RuCo@CNF	17270 /300	3.75/0.98	98.9	90 cycles (500 /1000)	[17]
RuO_2–CoTi LDO	5455 /100	3.2/0.6	N/A	60 cycles (166 /1000)	[18]
RuO_2–TiO_2@CT	16727 /250	-/1.05	N/A	238 cycles (250 /1000)	[19]

Note: Charge Potential (C.P.), Overpotential (O.P.), Coulombic Efficiency (CE), Multiwalled Carbon Nanotubes (MWCNTs), Ketjen Black (KB), Layered Double Oxide (LDO), Nanowire Arrays (NAs), Carbon Textiles (CT).

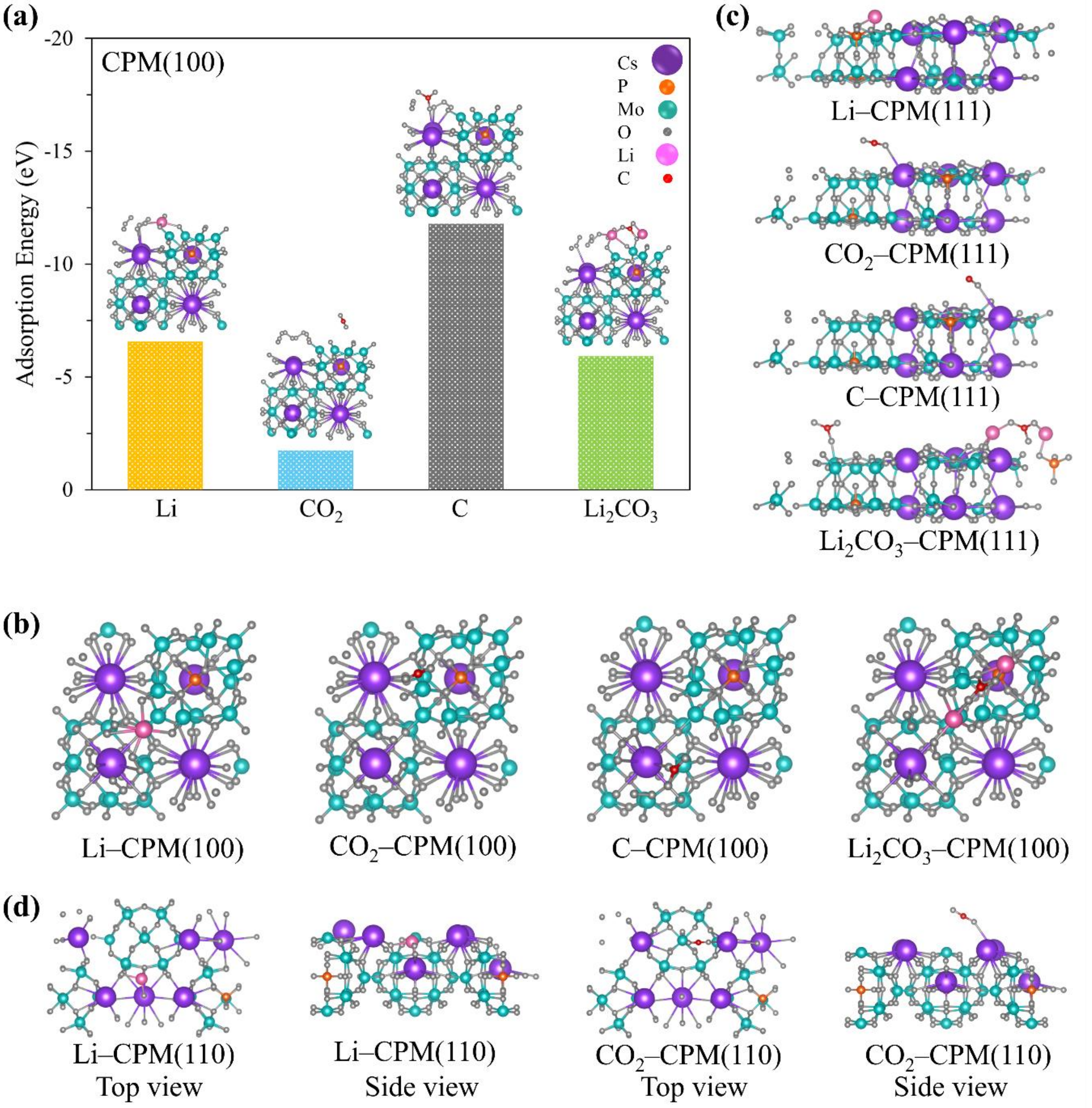

Figure S7. (a) Adsorption energies for Li, CO_2, C and Li_2CO_3 on the CPM(100) surface. Insets display the side view of Li, CO_2, C and Li_2CO_3 adsorption configurations on the CPM(100) surface. (b) The top view of Li, CO_2, C and Li_2CO_3 adsorption configurations on the CPM(100) surface. (c) The side view of Li, CO_2, C and Li_2CO_3 adsorption configurations on the CPM(111) surface. (d) Top and side views of Li and CO_2 adsorption configurations on the CPM(110) surface.

Table S2. Screening of different adsorption sites on the CPM(100) surface.

Adsorption Site	E_{ads}			
	Li	CO_2	Li_2CO_3	C
Cs–low O	-4.97	-1.55	-3.24	-6.81
Cs–high O	-5.46	–	-3.12	-11.78
Mo–P	-2.88	–	-4.83	-7.14
Bridge Cs–Mo	-6.57	-1.75	-5.92	-8.60

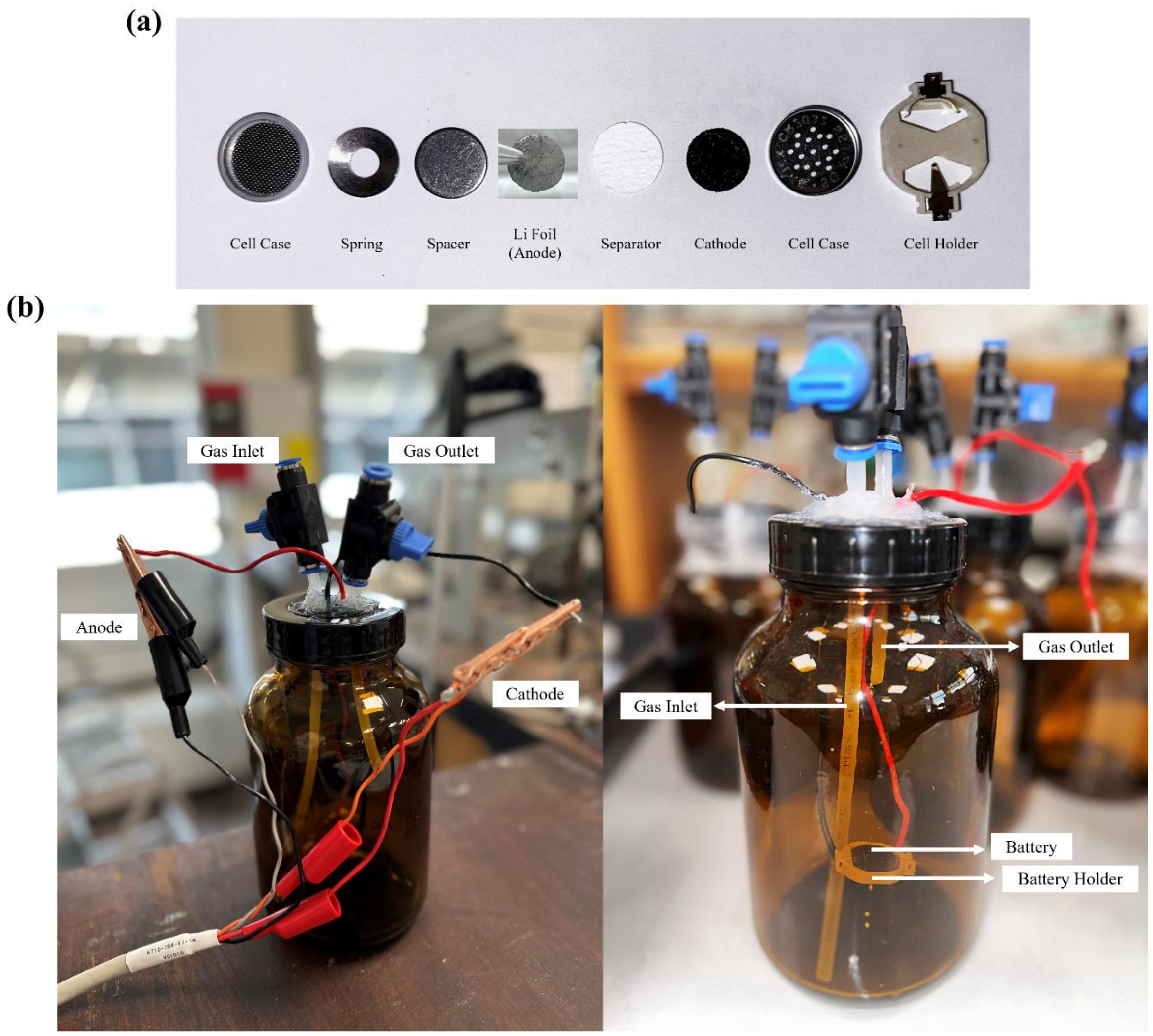

Figure S8. (a) Cell components of a Li–CO_2 battery. (b) A Li–CO_2 battery system composed of the assembled coin cell in a CO_2-filed homemade glass bottle.

References

[1] A. A. Coelho, *Coelho Software, Brisbane* **2007**.

[2] N. Paul, R. B. Hammond, T. N. Hunter, M. Edmondson, L. Maxwell, S. Biggs, *Polyhedron* **2015**, *89*, 129.

[3] A. Jain, S. P. Ong, G. Hautier, W. Chen, W. D. Richards, S. Dacek, S. Cholia, D. Gunter, D. Skinner, G. Ceder, K. A. Persson, *APL Mater.* **2013**, *1*, 11002.

[4] G. S. Pawley, *Appl. Crystallogr.* **1981**, *14*, 357.

[5] L. Marosi, E. Escalona Platero, J. Cifre, C. Otero Areán, *J. Mater. Chem.* **2000**, *10*, 1949.

[6] H. M. Rietveld, *Appl. Crystallogr.* **1969**, *2*, 65.

[7] S. Yang, Y. Qiao, P. He, Y. Liu, Z. Cheng, J. Zhu, H. Zhou, *Energy Environ. Sci.* **2017**, *10*, 972.

[8] S. Bie, M. Du, W. He, H. Zhang, Z. Yu, J. Liu, M. Liu, W. Yan, L. Zhou, Z. Zou, *ACS Appl. Mater. Interfaces* **2019**, *11*, 5146.

[9] K. M. Naik, A. K. Chourasia, M. Shavez, C. S. Sharma, *ChemSusChem* **2023**, *16*, e202300734.

[10] Y. Wang, J. Zhou, C. Lin, B. Chen, Z. Guan, A. M. Ebrahim, G. Qian, C. Ye, L. Chen, Y. Ge, Q. Yun, X. Wang, X. Zhou, G. Wang, K. Li, P. Lu, Y. Ma, Y. Xiong, T. Wang, L. Zheng, S. Chu, Y. Chen, B. Wang, C.-S. Lee, Y. Liu, Q. Zhang, Z. Fan, *Adv. Funct. Mater.* **2022**, *32*, 2202737.

[11] G. Wu, X. Li, Z. Zhang, P. Dong, M. Xu, H. Peng, X. Zeng, Y. Zhang, S. Liao, *J. Mater. Chem. A Mater.* **2020**, *8*, 3763.

[12] Z. Wang, B. Liu, X. Yang, C. Zhao, P. Dong, X. Li, Y. Zhang, K. Doyle-Davis, X. Zeng, Y. Zhang, X. Sun, *Adv. Funct. Mater.* **2023**, *33*, 2213931.

[13] X. Zhang, C. Wang, H. Li, X.-G. Wang, Y.-N. Chen, Z. Xie, Z. Zhou, *J. Mater. Chem. A Mater.* **2018**, *6*, 2792.

[14] X. Xiao, Z. Zhang, W. Yu, W. Shang, Y. Ma, X. Zhu, P. Tan, *ACS Appl. Energy Mater.* **2021**, *4*, 11858.

[15] W. Ma, S. Lu, X. Lei, X. Liu, Y. Ding, *J. Mater. Chem. A Mater.* **2018**, *6*, 20829.

[16] Y. Jin, F. Chen, J. Wang, R. L. Johnston, *Chem. Eng. J.* **2019**, *375*, 121978.

[17] Y. Jin, F. Chen, J. Wang, *ACS Sustain. Chem. Eng.* **2020**, *8*, 2783.

[18] S.-M. Xu, Z.-C. Ren, X. Liu, X. Liang, K.-X. Wang, J.-S. Chen, *Energy Storage Mater.* **2018**, *15*, 291.

[19] C. Wang, Y. Shang, Y. Lu, L. Qu, H. Yao, Z. Li, Q. Liu, *J. Power Sources* **2020**, *475*, 228703.
